# Exploration on the Interaction Ability of Antitumor Compound Bis-[2,6-difluoro-*N*-(hydroxyl-<κ>*O*)benzamidato-<κ>*O*]dibutylitin(IV) with Human Peroxisome Proliferator-Activated Receptor hPPAR*γ*

**DOI:** 10.1155/2018/3063271

**Published:** 2018-06-10

**Authors:** Jiaqi Mai, Yunlan Li, Xiaozhi Qiao, Xiaoqing Ji, Qingshan Li

**Affiliations:** ^1^School of Pharmaceutical Science, Shanxi Medical University, Taiyuan 030001, China; ^2^Shanxi University of Traditional Chinese Medicine, Jinzhong 030619, China

## Abstract

Diorganotin(IV) antitumor compound bis-[2,6-difluoro-*N*-(hydroxyl-<κ>*O*)benzamidato-<κ>*O*] (DBDF2,6T) was one of the novel patent organotin compounds with high antitumor activity and relatively low toxicity. In this study, several methods were used to study the interaction between DBDF2,6T and hPPAR*γ* protein, including fluorescence quenching, three-dimensional (3D) fluorescence, drug affinity responsive target stability (DARTS), ultrafiltration-LC, and molecular docking. According to the experimental results, the quenching process of the hPPAR*γ* protein was induced by static quenching mode to form a nonradiative ground-state complex with DBDF2,6T spontaneously, mainly through the hydrophobic force. DBDF2,6T could bind to the hPPAR*γ* protein directly and give the protein the ability of antienzymatic hydrolysis. And the binding mode of DBDF2,6T into hPPAR*γ* protein appeared to have an orientation towards residues of SER342 and GLY284. In conclusion, these methods could comprehensively reveal the interaction details of DBDF2,6T and the hPPAR*γ* protein and established a feasible way to preliminarily identify the agonist compounds for the hPPAR*γ* protein.

## 1. Introduction

Organotin compounds have many uses in our life, which could act as stabilizers in plastics, fungicides, industrial catalysts, and so on. [1]. Our research group had synthesized a series of organotin patent compounds which possessed high anticancer activity with low toxicity and devoted to clarify its mechanism of action [2]. From the results of proteomics data, these compounds might play the physiological role through the PPAR (peroxisome proliferator-activated receptor) signaling pathway, which was consistent with the reports that organotin compounds may function as endocrine-disrupting chemicals by affecting the function of the protein PPAR*γ* [3]. Consequently, a reasonable hypothesis was made to assume that these biologically active compounds might function through the PPAR signaling pathway as an agonist to the protein PPAR*γ* and further influence the expressions of the target genes.

PPARs proteins belong to the most important members of the nuclear receptor superfamily and can act as the ligand-activated transcription factors [4]. When the PPARs proteins bind to a specific ligand, the ligand-binding domains of PPARs will encounter the conformational change followed by promoting the recruitment of nuclear receptor coregulators such as steroid rector coactivator-1 (SRC-1) and eventually influence the transcription of downstream target genes [5]. The PPARs proteins have three isotypes which had been identified as PPAR*α*, PPAR*β*/*δ*, and PPAR*γ*. These three subtypes exhibit distinct tissue distributions and have unique biological functions [6, 7]. In particular, PPAR*γ* has received much focus these years for the important physiological functions played by its ligands. For example, thiazolidinediones (TZDs), a class of PPAR*γ* agonist compounds, had been used as a therapeutic compound for metabolic disorders such as type 2 diabetes and obesity [8], and it was also reported that the agonists to PPAR*γ* protein had a potential to be used as a new therapeutic approach to cancers, immune disorders, and so on. [9, 10]. Therefore, the experiments established to find ligands which could interact with the PPAR*γ* protein are promising works nowadays.

**Figure 1:**
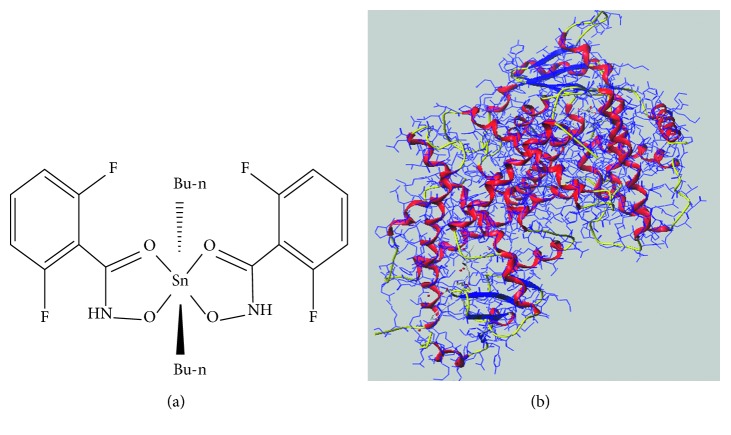
Structures of DBDF2,6T and hPPAR*γ* protein: (a) 2D structure of DBDF2,6T and (b) 3D structure of PPAR*γ* (4a4w.pdb).

In this study, a patent organotin compound DBDF2,6T (bis-[2,6-difluoro-*N*-(hydroxyl-<κ>*O*)benzamidato-<κ>*O*]dibutylitin) (patent number: CN200910074795.X and ZL01135148.9 (P)) which showed a high antitumor activity was assumed as a potential agonist. Several different methods were adopted to test and verify the interaction between DBDF2,6T and the hPPAR*γ* protein. Spectroscopic study, one of the most widely used methods for analyzing the interaction between small molecule and protein, was applied to provide parameters such as binding constants and types of interaction forces. DARTS and ultrafiltration-LC were used to verify such interaction while molecular docking was used to evaluate affinity between receptors and ligands in a theoretical way. These methods meet the requirements of low cost and high feasibility and perfectly supplement and verify each other, which could be used to find the new agonists of the hPPAR*γ* protein preliminarily and offered references for the interaction analysis between synthesized compounds and proteins. The structures of DBDF2,6T and the hPPAR*γ* protein are shown in Figure 1.

## 2. Materials and Methods

### 2.1. Reagent

DBDF2,6T was synthesized by Shanxi Medical University with purity over 99%. The protein human PPAR*γ* (hPPAR*γ*) was purchased from Flarebio Company (Flarebio Biotech LLC, Wu Han, China) and stored at −20°C. Pronase was purchased from Roche Diagnostic GmbH (Mannheim, Germany) and stored at 4°C. The Coomassie Blue R-250 was purchased from Sigma Company (Shanghai, China), and the PageRuler Prestained Protein Ladder was made by Thermo Fisher Scientific (Massachusetts, America). The solvent of organotin(IV) was configured by propanediol (Tianjin Fengchuan Chemical Reagent Science and Technology Co., Tianjin, China), ethylenediamine, and normal saline (Shijiazhuang Pharmaceutical, Shijiazhuang, China) (90 : 9 : 1, v/v/v). The disodium hydrogen phosphate dodecahydrate was purchased from Tianjin Fengchuan Chemical Reagent Company (Tianjin, China). The potassium phosphate monobasic was purchased from Tianjin Beichen Fangzheng Company (Tianjin, China).

All the other reagents used in this study were of analytical grade and were obtained commercially.

### 2.2. Fluorescence Quenching Spectrum

Amino acid residues such as tryptophan, tyrosine, and phenylalanine could empower the proteins with the ability to generate endogenous fluorescence. The fluorescence peaks of those three amino acids were located at 348 nm, 303 nm, and 282 nm, respectively. Actually, 95% of protein fluorescence was contributed to the tryptophan residue [11, 12]. Compared with other methods, fluorescence spectroscopy had many superior advantages including high sensitivity, selectivity, and easy operation [13]. Therefore, in this paper, the fluorescence quenching method was used to analyze the interaction between DBDF2,6T and the hPPAR*γ* protein.

The experiments were performed at 293 K and 310 K on a U-3900 spectrofluorophotometer (BaHens Instrument Co. Ltd., China). Protein hPPAR*γ* (10 *μ*g) was dissolved in a 2 mL PBS buffer. Several concentrations of DBDF2,6T (0.5 × 10^−6^, 1.0 × 10^−6^, 1.5 × 10^−6^, 2.5 × 10^−6^, 3.0 × 10^−6^, and 3.5 × 10^−6^ mol/L) were, respectively, incubated with the certain concentration of the hPPAR*γ* protein. Samples of protein hPPAR*γ* and hPPAR*γ*-DBDF2,6T complexes were measured in a 1 cm^2^ quartz cuvette. And the excitation and emission spectral slit widths were set as 10 nm. The emission spectra were recorded for light-scattering effects from 300 nm to 450 nm while the exciting wavelength was set as 280 nm.

### 2.3. Three-Dimensional Fluorescence Spectrum

The coordinate axes of the three-dimensional (3D) fluorescence spectrum were excitation wavelength, emission wavelength, and fluorescence intensity. It had been proved that the 3D fluorescence spectrum was an effective analytical technique to analyze the conformation changes of a protein in its solution state [14]. And this method could not only test the molecular structure change with much selectivity and sensitivity but also display fluorescent information of the sample solution comprehensively [15].

Experiments were performed at the temperature of 293 K. Protein hPPAR*γ* (10 *μ*g) was dissolved in a 2 mL PBS buffer and incubated with 2.0 × 10^−6^ mol/L DBDF2,6T for two minutes. Samples were tested on a U-3900 spectrofluorophotometer (BaHens Instrument Co. Ltd., China) with the parameters set as follows: excitation wavelength was from 200 nm to 300 nm; emission wavelength was from 320 nm to 450 nm; spectral slit width was 10 nm; and the gain value was 2.

### 2.4. DARTS with Pure hPPAR*γ* Protein

DARTS had been proved to be an efficient approach to efficiently verify drug-protein interactions when the protein was available in relatively pure form [16]. The basic principle of DARTS was that compounds were proposed to stabilize the combined protein globally or locally by reducing protease sensitivity of the target protein. This phenomenon was attributed to a specific conformational change caused by such a binding process, which would further induce protease recognition sites of the protein to be masked [17]. In this study, protein hPPAR*γ* regarded as target protein and pure hPPAR*γ* protein generated from recombinant plasmid were used in this experiment. Whether the presence of DBDF2,6T could reduce the proteolysis of the protein to validate the interaction between protein hPPAR*γ* and DBDF2,6T should be observed after incubating the hPPAR*γ* protein with DBDF2,6T.

Eight sample groups were set and divided into blank group, negative control group, and test group; each sample contained 0.5 *μ*g hPPAR*γ* protein. Except for the blank group, other samples were incubated with 2 *μ*L DMSO or 2 *μ*L DBDF2,6T with the concentration ranging from 1.0 × 10^−2^ mol/L to 1.0 × 10^−4^ mol/L for 60 min at 4°C and then digested with pronase (1 : 100) at room temperature for 30 min. The digestion was stopped by adding 5× SDS-PAGE sample loading buffer and boiling at 100°C for 10 min immediately. Samples were then subjected to electrophoresis on 8% SDS-PAGE. After electrophoresis, the gel was stained with Coomassie Brilliant Blue for 1 h and was observed after eluted overnight.

### 2.5. Ultrafiltration-Liquid Chromatography Experiment

Ultrafiltration-liquid chromatography (ultrafiltration-LC) was developed to verify the agonists of protein hPPAR*γ*. The main principle of ultrafiltration-LC was that the agonists of hPPAR*γ* had the ability to bind to the protein and would not be filtered out through the membrane of the ultrafiltration centrifuge tube, while after the protein denatured by dissolving in organic solvents, the compounds would be unbound to the protein and could be washed out through the membrane of the ultrafiltration centrifuge tube. Based on the ultraviolet absorption of the compound, the DBDF2,6T which were bound to hPPAR*γ* could be detected through analyzing the washed solution by liquid chromatography. This method was first used in such confirmatory experiment and marked by its simplicity, generality, and applicability [18].

The recombination protein hPPAR*γ* (20 *μ*g) was incubated with compound DBDF2,6T (2 *μ*L·10^−3^ mol/L) for 24 h at 4°C. After being filtered through a 10000 Da molecular weight cutoff ultrafiltration membrane (Millipore, UFC500396) by centrifugation at 13000 r/min for 8 min at 4°C, the sample was washed three times with 150 *μ*L PBS buffer (pH 7.4) and centrifuged at 13000 r/min for 12 min at 4°C to remove the unbond compounds. The washed solution was transferred to a new 10000 Da molecular weight cutoff ultrafiltration centrifuge tube and dissolved it in 400 *μ*L methanol. Centrifugation at 13000 r/min for 12 min was performed to wash out the compounds which were combined with the protein, and the washed solution was collected. Following reconstitution in 100 *μ*L of 50% aqueous methanol, the compound was analyzed using HPLC (Agilent Technologies). Denatured protein was used as a negative control, and in this experiment, the protein was heated at 98°C for 15 min to make it denatured.

The HPLC analysis was carried out using mobile phase methanol/0.5% phosphoric acid (28 : 72, v/v, pH 3.0) on a C18 column (Agilent TC-C18, 4.6 × 250 mm i.d., 5 *μ*m) at a flow rate of 0.8 mL/min and at 25°C. The detection wavelength was set at 264 nm.

### 2.6. Molecular Docking

Surflex-Dock, docking module in SYBYL software (UCSF), was performed to determine the binding model of protein hPPAR*γ* (4a4w.pdb) and DBDF2,6T. It used prototype molecule (protomal) to represent protein binding pocket, utilizing probe to test the qualities of protein pocket such as surface hydrophobicity and could generate the invert transform of an active protein pocket. This method had high docking accuracy and could be used to research on the interaction between biomacromolecules and small molecular ligands [19].

Sybly × 2.0 was used to draw two-dimensional structure of DBDF2,6T with standard bonds and angles. In the process of optimizing the compound structure, minimize details and parameters of modify were set as follows. Minimize details: the iterations were set as 10000, and the color option was set as force. Parameters of modify: the force field was set as Tripos and the charges were set as Gasteiger–Marsili. In the docking process, A/YFB99 was chosen as extracted ligand structure and hydrogen molecules were added to the protein, and the modify details were set as follows: the force field was AMBER7 FF99 and the charge was AMBER. All other parameters were used the default value of SYBYL during the protein pocket generation and the molecular docking.

## 3. Results and Discussions

### 3.1. Fluorescence Spectrum of Protein hPPAR*γ* and hPPAR*γ*-DBDF2,6T Complexes

**Figure 2:**
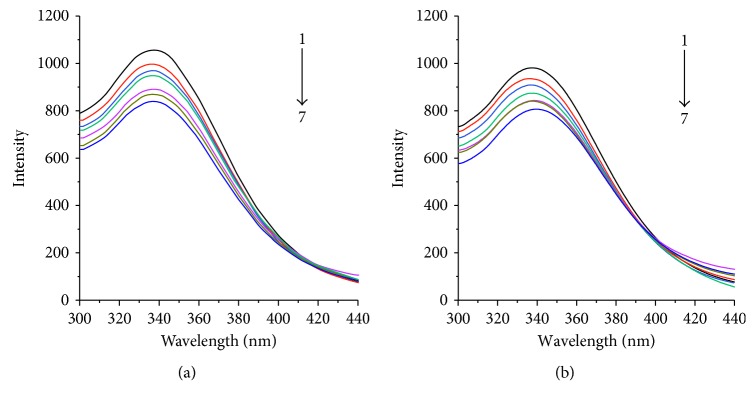
Fluorescence quenching spectra of protein hPPAR*γ* with series concentrations of DBDF2,6T at different temperatures: (a) 293 K and (b) 310 K. DBDF2,6T(a→j): 0, 0.5 × 10^−6^, 1.0 × 10^−6^, 1.5 × 10^−6^, 2.5 × 10^−6^, 3.0 × 10^−6^, and 3.5 × 10^−6^ mol/L; hPPAR*γ* protein: 5 *μ*g/mL.

Fluorescence curves of the protein hPPAR*γ* and hPPAR*γ*-DBDF2,6T complexes are shown in Figure 2. The experiments were carried out in two temperatures with other experimental conditions unchanged. It can be observed from Figure 2 that, in both the experimental system of 273 K and 310 K, the intensity of fluorescence of the hPPAR*γ* protein was decreased while increasing the concentration of DBDF2,6T which was incubated with the hPPAR*γ* protein. And the highest fluorescent intensity values of hPPAR*γ* were recorded for next step of theoretical calculation.

### 3.2. Mechanism of Fluorescence Quenching

While the pH, temperature, and ionic strength were kept as constants, the types of fluorescence quenching could be classified into two categories: dynamic quenching and static quenching [20]. Dynamic quenching was caused by the fluorescent chromophore interacted with a quencher in excitation state, while the causes of static quenching were of three types: the first one, the fluorescent chromophore interacted with a quencher in ground state and came into being a nonfluorescent compound; the second one, the medium near the fluorescent chromophore had a polarity change, which caused by the conformational change of the protein attributing to the combination with the quencher; and the third one, a radiationless energy transfer between the fluorescent chromophore and the quencher [21].

The dynamic quenching obeyed the Stern–Volmer equation, and the formulas are shown as follows:

(1) $$\begin{array}{c} \frac{F_{0}}{F}=1+K_{\text{q}}\tau_{0}c\left[Q\right]=1+K_{\text{SV}}c\left[Q\right],\\ K_{\text{q}}=\frac{K_{\text{SV}}}{\tau_{0}}, \end{array}$$

where *K*_SV_ is the Stern–Volmer quenching constant, *K*_q_ is the bimolecular quenching constant, and *τ*_0_ is the average lifetime of the molecule which always be considered as 1.0 × 10^−8^ s [22, 23]. *c*[*Q*] is the concentration of the quencher. *F* and *F*_0_ correspondingly represent the intensity of the fluorescence of the protein added with the quencher or not.

The Stern–Volmer quenching curves were drawn according to the data obtained from fluorescence quenching spectra, and the corresponding linear regression equations and correlation coefficients are shown in Table 1.

**Figure 3:**
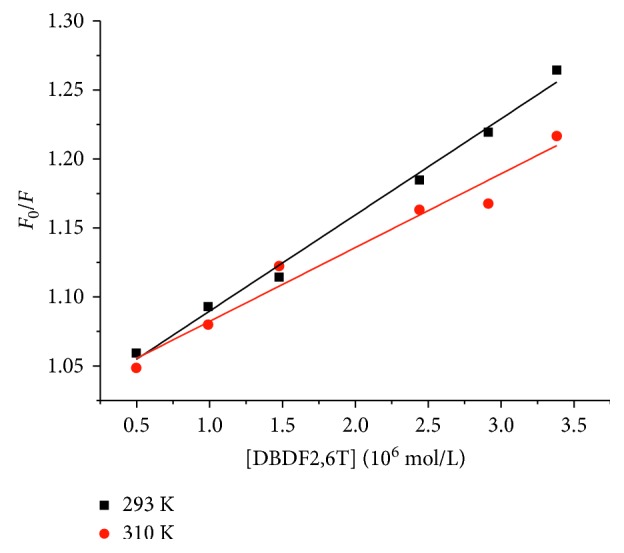
Stern–Volmer plots of hPPAR*γ* interacted with DBDF2,6T at different temperatures.

When a quencher interacted with a biomacromolecule, the maximum value of diffusion collision rate constant was considered as 2.0 × 10^10^ L/mol/s. According to the computing results shown in Table 1, the dynamic quenching constant (*K*_q_) between DBDF2,6T and the hPPAR*γ* protein was of the order of magnitude of 10^12^, which was much bigger than the maximum value of diffusion collision rate constant. Consequently, the type of fluorescence quenching of the hPPAR*γ* protein induced by DBDF2,6T was preliminary defined as a kind of static quenching [24]. In dynamic quenching, which was associated with diffusion, quenching constant of fluorescent material was increasing as the temperature increased. But from the Figure 3, the slope of the Stern–Volmer lines was decreased while increasing the temperature of the experimental system, which further confirmed the quenching mechanism of DBDF2,6T with the hPPAR*γ* protein was static quenching.

### 3.3. Binding Constants and Binding Site Numbers

**Figure 4:**
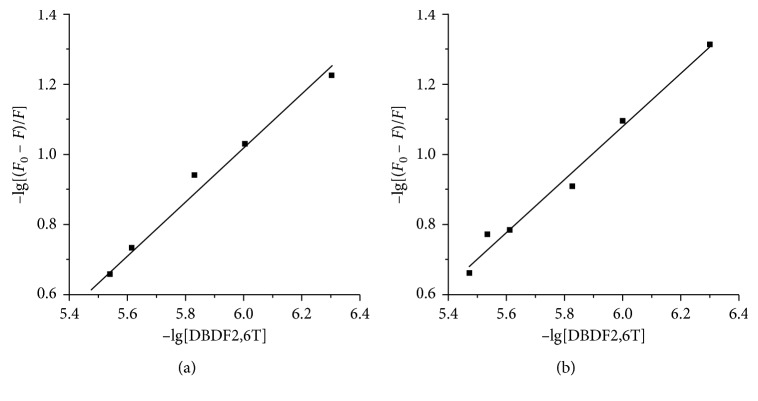
A plot of −lg[*F*_0_ − *F*/*F*] versus −lg[DBDF2,6T] at different temperatures: (a) 293 K and (b) 310 K.

Equation (2) is the Lineweaver–Burk double-reciprocal equation, and (3) was deduced by (2) [25]:

(2) $$\begin{array}{c} \frac{1}{F_{0}-F}=\frac{1}{F_{0}}+\frac{1}{K_{\text{A}}F_{0}c\left[Q\right]}, \end{array}$$

(3) $$\begin{array}{c} \mathrm{lg}\left[\frac{F_{0}-F}{F}\right]=\mathrm{lg}K_{\text{A}}+\text{nlgc}\left[\text{Q}\right], \end{array}$$

where *K*_A_ is the binding constant and *n* is the number of independent binding sites. When −lg[*F*_0_ − *F*/*F*] were plotted against lgc(*Q*), a straight line could be drawn and is shown in Figure 4. The corresponding computing results are shown in Table 2.

In both the experimental temperatures (273 K and 310 K), the computing binding site numbers were near to 1, which meant that the hPPAR*γ*-DBDF2,6T complexes were formed by protein hPPAR*γ* and DBDF2,6T at the ratio approximately to 1 : 1. And the binding constants were of the order of magnitude of 10^3^, which meant that the binding ability between them was pretty strong.

### 3.4. Thermodynamic Parameters and Interaction Forces

The interaction forces between small molecules and biomacromolecules were belonged to noncovalent force including hydrogen bond, van der Waals force, electrostatic attraction, and so on. The main acting force between hPPAR*γ* protein and DBDF2,6T could be judged according to the thermodynamic parameters which were calculated based on the Van't Hoff equation [26]. From the previous researches on interaction abilities, it was assumed that different proteins and compounds had different main acting force [27]. The thermodynamic parameters were calculated according to following equations:

(4) $$\begin{array}{c} \Delta H=\frac{2.303RT_{1}T_{2}}{T_{2}-T_{1}}\mathrm{lg}\frac{K_{2}}{K_{1}}, \end{array}$$

(5) $$\begin{array}{c} \Delta G=-RT\ln K, \end{array}$$

(6) $$\begin{array}{c} \Delta S=\frac{\Delta H-\Delta G}{T}, \end{array}$$

where *R* is the gas constant, Δ*G* is the Gibbs free energy change, Δ*S* is the entropy change, Δ*H* is the enthalpy change, and *K* is the Stern–Volmer quenching constant. Δ*H* could be considered as a constant when the temperature changed in small range. And the Ross law indicated that, if Δ*H* > 0 and Δ*S* > 0, the main acting force between small molecules and biomacromolecules would be hydrophobic force; if Δ*H* < 0 and Δ*S* < 0, it would be hydrogen bond and van der Waals force; and if Δ*H* ≈ 0 and Δ*S* > 0, it would be electrostatic force [28].

According to the computing results shown in Table 3, Δ*H* was 19.03 kJ/mol, Δ*S* was 3.92 J/mol·K and 3.90 J/mol·K correspondingly at 293 K and 310 K, and Δ*G* was −20.18 kJ/mol and −20.24 kJ/mol correspondingly at 293 K and 310 K. Based on the Ross law, the main acting force between hPPAR*γ* protein and DBDF2,6T was hydrophobic force. In addition, the regulator effect of several kinds of interaction forces and relevant microenvironments were both responsible for the macroscopic consequence [29].

### 3.5. Conformational Change of hPPAR*γ* Protein

**Figure 5:**
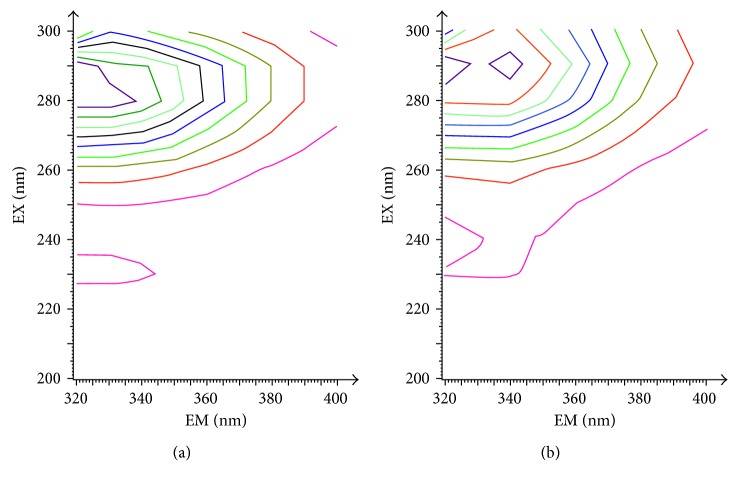
The contour maps of two florescent systems: (a) hPPAR*γ* and (b) hPPAR*γ*-DBDF2,6T system (*T* = 293 K).

The 3D fluorescence spectrum is shown in Figure 5 in the form of intensive contour map. The related data are shown in Table 4.

It can be observed from Figure 5 that two typical fluorescence peaks of proteins were located approximately at *λ*_em_ = 340 nm. In order to observe the peaks of fluorescent groups more clearly, the excitation wavelength range was set smaller than emission wavelength range, so the spectra of Rayleigh scattering were not available on the picture [19]. After the hPPAR*γ* protein was incubated with DBDF2,6T, the location of both fluorescence peaks did not show a significant change, but the intensity of each peak was reduced at different degrees. From the 3D fluorescence spectra of the hPPAR*γ* protein (Figure 5(a)), the intensity ratio of the big peak to the small one was 7.97 : 1, while after the protein was incubated with DBDF2,6T (Figure 5(b)), the value was changed to 8.00 : 1, and the DBDF2,6T showed a strong quenching effect on the big peak which was located at about 290/340 (*λ*_ex_/*λ*_em_). The 3D fluorescence spectrum indicated a conformational change of the specific structures of the hPPAR*γ* protein, which could further validate the interaction between the hPPAR*γ* protein and DBDF2,6T [30].

### 3.6. Confirmation of the Interaction Ability Using DARTS Technique

To identify the binding targets for small molecules, the key advantage of DARTS method was no sample pretreatments such as labeling the ligand [17]. And the method was particularly useful when a compound had a lower affinity with the target, even the binding constant was in micromolar range [31]. To confirm the feasibility of the DARTS applying to the hPPAR*γ* protein, a preliminary experiment had been performed to research the digestion effects of the protease on the hPPAR*γ* protein. And the pronase was used for digestion because it had been proved to be more useful for DARTS than any other protease [16]. In preliminary experiment, the digestion effects of time of enzymolysis and the concentration of pronase had been investigated.

**Figure 6:**
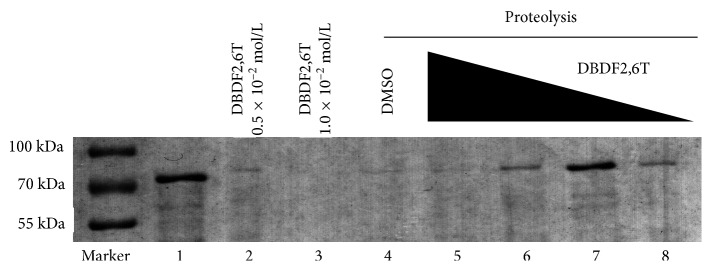
DARTS detection via SDS-PAGE. Band 1 was pure hPPAR*γ* protein without incubation with DBDF2,6T or proteolysis; bands 2 and 3 were hPPAR*γ* protein incubated with 0.5 × 10^−2^ mol/L and 1.0 × 10^−2^ mol/L DBDF2,6T, respectively, and without proteolysis; bands 4 to 8 were hPPAR*γ* protein incubated with DMSO and different concentrations of DBDF2,6T (5→8: 0.5 × 10^−2^ mol/L, 0.5 × 10^−3^ mol/L, 0.5 × 10^−4^ mol/L, and 0.5 × 10^−5^ mol/L), respectively, and with proteolysis.

After electrophoresis and staining, the protein bands of each sample are shown in Figure 6. Compared with the DMSO control, under certain conditions, the antienzymatic hydrolysis ability of hPPAR*γ* protein did exist and was closely related to the concentration of DBDF2,6T incubated with the protein. In 0.5 × 10^−4^ mol/L DBDF2,6T, the strongest antienzymatic hydrolysis ability of the hPPAR*γ* protein would appear, and such ability could be weakened with the change in the concentration. Although DBDF2,6T had shown hydrolysis ability to the hPPAR*γ* protein at relatively high concentration, the protective functions of DBDF2,6T to the hPPAR*γ* protein still could be observed and existed concentration-effect relationships in some extent. Consequently, the interaction between hPPAR*γ* protein and DBDF2,6T could be indirectly verified [32].

### 3.7. Confirmation of the Binding Ability Using Ultrafiltration-LC Technique

**Figure 7:**
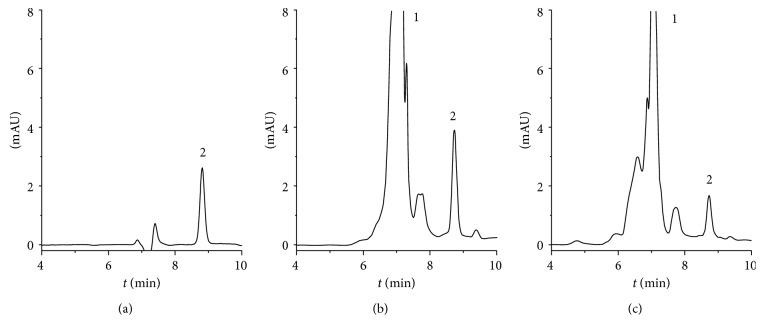
The results of ultrafiltration-LC experiment: (a) compound DBDF2,6T dissolved in 50% aqueous methanol; (b) the sample which was prepared using natural protein; (c) the negative control which was prepared using denatured protein. 1: the peak of impurities from solvent of protein hPPAR; 2: the peak of compound DBDF2,6T.

The results of ultrafiltration-LC experiment are shown in Figure 7. It can be observed from the chromatograms that both the sample and the negative control had an obvious peak at location about 8.75 min which belongs to compound DBDF2,6T. Significant signal enhancement of the peak of compound DBDF2,6T between the sample and the negative control indicated a specific binding between DBDF2,6T and the recombinant protein hPPAR*γ*, while the signal of DBDF2,6T in the negative control was attributed to the nonspecific binding [33]. The big impurity peak was located at about 7 min attributed to the solution of recombination protein hPPAR*γ*. Therefore, the experiment of ultrafiltration-LC did verify that compound DBDF2,6T could bind to pure protein hPPAR*γ* directly in physiological environment [34].

### 3.8. Exploration of the Theoretical Binding Details Using Molecular Docking

**Figure 8:**
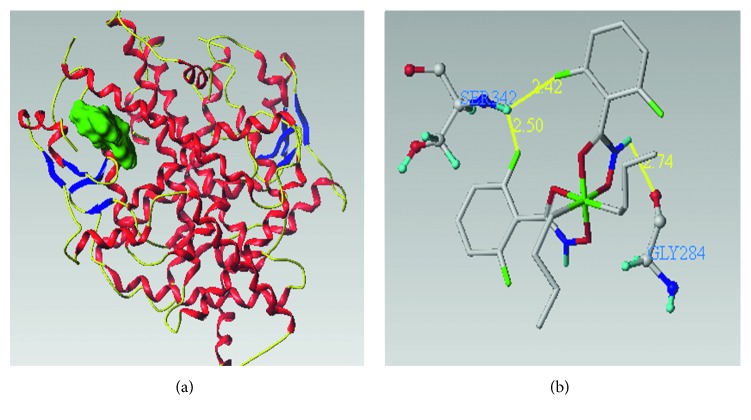
The docking results of DBDF2,6T to hPPAR*γ* protein: (a) the generated pocket of hPPAR*γ* protein; (b) hydrogen bond graph of DBDF2,6T to the interacted residues of the hPPAR*γ* protein. The yellow lines in the picture represent the hydrogen bond.

In order to further understand the interaction between DBDF2,6T and hPPAR*γ* protein, molecular docking was used to explore the theoretical binding details of them [35]. Among the docking of 12 conformers of DBDF2,6T to generate pocket of the hPPAR*γ* protein, the highest total score was 7.14 and the corresponding crash score and polar score were −1.86 and 0.00, respectively, which meant that DBDF2,6T had a pretty strong affinity to hPPAR*γ* protein, and such docking process was under a relatively comfortable level of molecules [36]. The generated pocket of hPPAR*γ* is shown in Figure 8(a), and the hydrogen bond graph is shown in Figure 8(b). In conclusion, DBDF2,6T could theoretically bind to hPPAR*γ* protein with pretty strong binding strength, and it could directly interact with SER342 and GLY284 of hPPAR*γ* protein by hydrogen bond. The hydrogen bond lengths between DBDF2,6T and SER342 were 2.50 Å and 2.42 Å, and that between DBDF2,6T and GLY284 was 2.74 Å.

## 4. Conclusions

This study analyzed the interaction between the novel patent organotin compound DBDF2,6T and the hPPAR*γ* protein under physiological condition with the methods of fluorescence quenching, 3D fluorescence, DARTS, ultrafiltration-LC, and computer molecular docking. According to the spectroscopic experimental data, DBDF2,6T could interact with the hPPAR*γ* protein and formed a nonradiative ground-state complex of hPPAR*γ*-DBDF2,6T, mainly through hydrophobic force. Such a reaction was spontaneous and could cause a conformational change of the hPPAR*γ* protein. And the experiments of DARTS and ultrafiltration-LC preliminarily proved the possibility of DBDF2,6T to be an agonist compound to hPPAR*γ* protein. Considering the anticancer activity of DBDF2,6T and various physiological functions performed by agonists of PPAR*γ* protein, the conclusion could be drawn that DBDF2,6T had a possibility to interact with the hPPAR*γ* protein as an agonist and finally inducing physiological effects such as anticancer activity. This work successfully revealed the interaction of DBDF2,6T with hPPAR*γ* protein and established a feasible way to validate the agonist compounds for hPPAR*γ* protein.

## Figures and Tables

**Table 1:** Linear regression equations and Stern–Volmer quenching rate constants at different temperatures.

Temperature *T* (K)	Linear regression equation	Correlation coefficient (*r*)	Dynamic quenching constant (*K*_q_/L·mol^−1^·s^−1^)
293	*F*_0_/*F* = 0.0697 × 10^6^*c*[*Q*] + 1.0201	0.9926	6.97 × 10^12^
310	*F*_0_/*F* = 0.0535 × 10^6^*c*[*Q*] + 1.0288	0.9679	5.35 × 10^12^

**Table 2:** Binding constants and the numbers of binding sites of hPPAR*γ* protein with DBDF2,6T at different temperatures.

Temperature *T* (K)	Numbers of binding sites	Binding constant, *K*_A_ (L/mol)	Correlation coefficient (*r*^2^)
293	0.77	3.96 × 10^3^	0.9841
310	0.75	2.58 × 10^3^	0.9856

**Table 3:** Thermodynamic parameters of reaction system of hPPAR*γ* protein and DBDF2,6T.

Temperature *T* (K)	Δ*H* (kJ/mol)	Δ*S* (J/mol·K)	Δ*G* (kJ/mol)
293	19.03	3.92	−20.18
310		3.90	−20.24

**Table 4:** Several characteristic parameters of 3D fluorescence experiments.

System and parameters	PPAR*γ*		PPAR*γ* + DBDF2,6T	
Fluorescence peak	Peak 1	Peak 2	Peak 1	Peak 2
Peak position (*λ*_ex_/*λ*_em_ nm/nm)	283/337	230/328	290/340	231/332
Relative intensity (*I*)	1275	160	1200	150
*I*_1_/*I*_2_	7.97 : 1		8.00 : 1	
Stokes shift (Δ*λ*/nm)	48	107	50	109

## Data Availability

The data used to support the findings of this study are available from the corresponding author upon request.

## Abbreviations

DBDF2,6T: Bis-[2,6-difluoro-*N*-(hydroxyl-<κ>*O*)benzamidato-<κ>*O*]dibutylitin
hPPAR*γ*: Human peroxisome proliferator-activated receptor gamma
3D fluorescence: Three-dimensional fluorescence
DARTS: Drug affinity responsive target stability
LC: Liquid chromatography.

## References

[1] Okoro H. K., Fatoki O. S., Adekola F. A., Ximba B. J., Snyman R. G., Opeolu B. (2011). Human exposure, biomarkers, and fate of organotin in the environment. *Reviews of Environmental Contamination and Toxicology*.

[2] Yan C., Zhang J., Liang T., Li Q. (2015). Diorganotin(IV) complexes with 4-nitro-N-phthaloyl-glycine: synthesis, characterization, antitumor activity and DNA-binding studies. *Biomedicine and Pharmacotherapy*.

[3] le Maire A., Grimaldi M., Roecklin D. (2009). Activation of RXR–PPAR heterodimers by organotin environmental endocrine disruptors. *EMBO Reports*.

[4] Cho M. C., Lee D. H., Kim E. J. (2011). Novel PPAR*γ* partial agonists with weak activity and no cytotoxicity; identified by a simple PPAR*γ* ligand screening system. *Molecular and Cellular Biochemistry*.

[5] Chen S., Johnson B. A., Li Y. (2000). Both coactivator LXXLL motif-dependent and–independent interactions are required for peroxisome proliferator-activated receptor gamma (PPARgamma) function. *Journal of Biological Chemistry*.

[6] Cho M. C., Yoon H. E., Kang J. W. (2006). A simple method to screen ligands of peroxisome proliferator-activated receptor delta. *European Journal of Pharmaceutical Sciences*.

[7] Kadivar A., Khoei H. H., Hassanpour H. (2016). Peroxisome proliferator-activated receptors (PPAR*α*, PPAR*γ* and PPAR*β*/*δ*) gene expression profile on ram spermatozoa and their relation to the sperm motility. *Veterinary Research Forum*.

[8] Houseknecht K. L., Cole B. M., Steele P. J. (2002). Peroxisome proliferator-activated receptor gamma (PPARgamma) and its ligands: a review. *Domestic Animal Endocrinology*.

[9] Vella V., Nicolosi M. L., Giuliano S., Bellomo M., Belfiore A., Malaguarnera R. (2017). PPAR-*γ* agonists as antineoplastic agents in cancers with dysregulated IGF axis. *Frontiers in Endocrinology*.

[10] Xu Z., Wang G., Zhu Y. (2018). PPAR-*γ* agonist ameliorates liver pathology accompanied by increasing regulatory B and T cells in high-fat-diet mice. *Obesity*.

[11] Tian Z., Zang F., Luo W. (2015). Spectroscopic study on the interaction between mononaphthalimide spermidine (MINS) and bovine serum albumin (BSA). *Journal of Photochemistry and Photobiology B: Biology*.

[12] Khan M. M., Tayyab S. (2011). Understanding the role of internal lysine residues of serum albumins in conformational stability and bilirubin binding. *Biochimica et Biophysica Acta (BBA)-Protein Structure and Molecular Enzymology*.

[13] Gelamo E. L., Tabak M. (2000). Spectroscopic studies on the interaction of bovine (BSA) and human (HSA) serum albumins with ionic surfactants. *Spectrochimica Acta Part A: Molecular and Biomolecular Spectroscopy*.

[14] Borissevitch L. E., Tominaga T. T., Imasato H. (1999). Fluorescence and optical absorption study of interaction of two water soluble porphyrins with bovine serum albumin. The role of albumin and porphyrin aggregation. *Journal of Luminescence*.

[15] Liu X. J., Wu X. Y., Qi C. Y., Cui J. S. (2012). Applications of three-dimensional fluorescent spectroscopy analysis technology. *Heibei Journal of Industrial and Technology*.

[16] Pai M. Y., Lomenick B., Hwang H. (2015). Drug affinity responsive target stability (DARTS) for small-molecule target identification. *Methods in Molecular Biology*.

[17] Derry M. M., Somasagara R. R., Raina K. (2014). Target identification of grape seed extract in colorectal cancer using drug affinity responsive target stability (DARTS) technique: role of endoplasmic reticulum stress response proteins. *Current Cancer Drug Targets*.

[18] Choi Y., Jung Y., Kim S.-N. (2015). Identification of eupatilin from Artemisia argyi as a selective PPARα agonist using affinity selection ultrafiltration LC-MS. *Molecules*.

[19] Wei Y., Niu L., Liu X. (2016). Spectroscopic studies and molecular docking on the interaction of organotin antitumor compound bis[2,4-difluoro-N-(hydroxy-<κ>O)benzamidato-<κ>O]diphenyltin(IV) with human cytochrome P450 3A4 protease. *Spectrochimica Acta Part A: Molecular and Biomolecular Spectroscopy*.

[20] Brown M. P., Royer C. (1997). Fluorescence spectroscopy as a tool to investigate protein interactions. *Current Opinion in Biotechnology*.

[21] Chen G. Z., Huang Z. X., Xu J. G. (1990). *Fluorescence Spectrometry*.

[22] Shaikh S. M. T., Seetharamappa J., Ashoka S., Kandagal P. B. (2007). A study of the interaction between bromopyrogallol red and bovine serum albumin by spectroscopic methods. *Dyes and Pigments*.

[23] Sun W., Du Y., Chen J., Kou J., Yu B. (2009). Interaction between titanium dioxide nanoparticles and human serum albumin revealed by fluorescence spectroscopy in the absence of photoactivation. *Journal of Luminescence*.

[24] Kathiravan M., Chandramohan R., Renganathan S., Sekar S. (2009). Spectroscopic studies on the interaction between phycocyanin and bovine serum albumin. *Journal of Molecular Structure*.

[25] Bi S., Song D., Tian Y., Zhou X., Liu Z., Zhang H. (2005). Molecular spectroscopic study on the interaction of tetracyclines with serum albumins. *Spectrochimica Acta Part A: Molecular and Biomolecular Spectroscopy*.

[26] Zhang L. N., Chen X., Xia Y. (2009). Study on interaction mechanism between meso-tetra–(4-hydroxyphenyl)-Zn porphyrin and bovine serum albumin by fluorescence method. *Spectroscopy and Spectral Analysis*.

[27] Gao L., Cao H. Y., Liu G. (2012). Spectroscopy on the interaction between four metalloporphyrin complexes and serum albumin. *Chemical Reagents*.

[28] Ganjali M. R., Faridbod F., Divsalar A. (2010). Nano-composite carbon pate electrode used for biophysical study of Ho3 ion interaction with human serum albumin V. *International Journal of Electrochemical Science*.

[29] Gonzalez-Jimencz J., Jacquotte H., Cayre J. (1992). Fluorescence quenching studies on binding fluoreno-9-spiro-oxazolidinedione to human serum albumin. *Chemico-Biological Interactions*.

[30] Zhang Q. Q., Lei S. H., Wang X. L., Wang L., Zhu C.-J. (2006). Discrimination of phytoplankton classes using characteristic spectra of 3D fluorescence spectra. *Spectrochimica Acta Part A Molecular and Biomolecular Spectroscopy*.

[31] Dal Piaz F., Vera Saltos M. B., Franceschelli S. (2016). Drug affinity responsive target stability (DARTS) identifies laurifolioside as a new clathrin heavy chain modulator. *Journal of Natural Products*.

[32] Rodriguez-Furlan C., Zhang C., Raikhel N. (2017). Drug affinity responsive target stability (DARTS) to resolve protein–small molecule interaction in arabidopsis. *Current Protocols in Plant Biology*.

[33] Tang P., Si S., Liu L. (2015). Analysis of bovine serum albumin ligands from *Puerariae flos* using ultrafiltration combined with HPLC-MS. *Journal of Chemistry*.

[34] Liu Z., Lin Z., Chen S., Wang L., Xian S. (2017). Rapid screening of potential phosphodiesterase inhibitors from the roots of *Ilex pubescens* hook. et arn. Using a combination of ultrafiltration and LC-MS. *Evidence-Based Complementary and Alternative Medicine*.

[35] Hou G., Zhang R., Hao X., Liu C. (2017). An exploration of the effect and the interaction mechanism of bisphenol A on waste sludge hydrolysis with multi-spectra, isothermal titration microcalorimetry and molecule docking. *Journal of Hazardous Materials*.

[36] Spitzer R., Jain A. N. (2012). Surflex-dock: docking benchmarks and real-world application. *Journal of Computer-Aided Molecular Design*.

